# Supplementary material for: Budget Impact Analysis of Adopting a One-Step Nucleic Acid Amplification Testing (NAAT) Alone Diagnostic Pathway for Clostridioides difficile in Japan Compared to a Two-Step Algorithm with Glutamate Dehydrogenase/Toxin Followed by NAAT
Source: Diagnostics (Basel). 2023 Apr 18;13(8):1463. doi: 10.3390/diagnostics13081463 (PMC10137341; doi:10.3390/diagnostics13081463)
Supplement: Supplementary file 1 [file diagnostics-13-01463-s001.zip › diagnostics-2241345-supplementary.pdf]

Supplementary Table S1: One-way sensitivity analysis of input parameters

Data inputs		Budget impact (JPY)		Cost per true positive CDI diagnosed (JPY)	
**Clinical parameters (proportion)**	**Base case (range)**	**Lower bound**	**Upper bound**	**Lower bound**	**Upper bound**
Prevalence of CDI	0.272 (0.238-0.308)	$286,019,903	$161,884,578	- $22,399	-$29,222
CDI associated mortality	0.0690 (0.0590-0.0790)	$222,592,923	$229,166,211	- $26,468	-$25,827
Cure from CDI treatment	0.788 (0.746-0.827)	$225,989,391	$225,886,360	- $26,386	-$26,146
Cure from recurrence (vancomycin taper and pulsed)	0.690 (0.619-0.761)	$225,512,346	- $26,327	- $25,966	$226,260,374
Cure from intravenous metronidazole and vancomycin due to treatment failure	0.841 (0.733-0.949)	$225,828,975	- $26,150	- $26,146	$226,017,159
Probability of first recurrence of CDI	0.126 (0.120-0.132)	$226,295,761	- $25,822	- $26,471	$225,476,959
Probability of second recurrence of CDI	0.227 (0.205-0.251)	$225,983,605	- $25,983	- $26,324	$225,797,219
CDI associated mortality due to undertreatment	0.138 (0.118-0.158)	$232,570,745	- $26,420	- $25,873	$219,201,975
Increase in mortality due to treatment failure	3.90 (1.40-10.7)	$221,744,608	- $26,260	- $25,902	$227,824,419
Increase in mortality due to recurrent CDI	1.00 (0.750-1.25)	$225,969,489	$225,803,231	- $26,228	-$26,065
Probability of colectomy from treatment failure	0.0240 (0.00500-0.0685)	$222,603,363	$233,245,473	- $25,977	-$26,526
**Diagnostic parameters**	**Range**	**Lower bound**	**Upper bound**	**Lower bound**	**Upper bound**
NAAT sensitivity	0.981 (0.899-1.00)	$459,483,312	$171,760,237	$7,573	- $32,809
NAAT specificity	0.989 (0.960-0.990)	$915,560,576	$202,104,490	- $407	-$27,034
GDH sensitivity	0.925 (0.818-0.979)	- $316,592,015	$499,660,493	- $119,827	$13,357
GDH specificity	0.944 (0.899-0.973)	$198,673,994	$243,423,218	- $27,238	- $25,443
Toxin antigen sensitivity	0.528 (0.386-0.667)	$196,896,464	$254,263,793	- $28,754	- $23,608
Toxin antigen specificity	1.00 (0.979-1.00)	$196,970,944	$225,886,360	- $27,306	- $26,146
**Economic parameters**	**Range**	**Lower bound**	**Upper bound**	**Lower bound**	**Upper bound**
Cost of managing recurrent CDI	659,923 (259,539-432,566)	$223,692,172	$228,080,548	- $24,641	- $27,651
Cost of managing initial CDI	346,053 (494,942-824,903)	$247,797,261	$203,975,459	- $18,833	- $33,459
Cost of NAAT	4,500 (3,375-5,625)	$133,281,843	$318,490,877	- $29,563	- $22,730
Cost of GDH/toxin	800 (600-1,000)	$245,886,360	$205,886,360	- $25,344	- $26,948
Cost of laparoscopic colectomy	1,705,371 (1,699,709-1,711,033)	$225,869,067	$225,903,653	- $26,143	- $26,149
Multiplier for cost of managing false negatives	1.57 (1.18-1.97)	$465,801,920	- $14,029,200	- $16,341	- $35,952

CDI: Clostridioides difficile infection; **GDH**: glutamate dehydrogenase; JPY: Japanese Yen; **NAAT**: Nucleic Acid Amplification Test
